# Supplementary material for: Six-electron-conversion selenium cathodes stabilized by dead-selenium revitalizer for aqueous zinc batteries
Source: Nat Commun. 2025 Apr 18;16:3707. doi: 10.1038/s41467-025-58859-3 (PMC12008412; doi:10.1038/s41467-025-58859-3)
Supplement: Supplementary file 1 — Supplementary Information [file 41467_2025_58859_MOESM1_ESM.pdf]

Six-electron-conversion selenium cathodes stabilized by dead-selenium revitalizer for aqueous zinc batteries

Jingwei Du[1], Jiaxu Zhang[1], Xingyuan Chu[1], Hao Xu[1], Yirong Zhao[2], Markus Löffler[3], Gang Wang[1,4], Dongqi Li[1], Quanquan Guo[5], Ahiud Morag[1,5], Jie Du[1], Jianxin Zou[6], Daria Mikhailova[7], Vlastimil Mazánek[8], Zdeněk Sofer[8], Xinliang Feng[1, 5, *], Minghao Yu[1, 5, *]

[1]Faculty of Chemistry and Food Chemistry & Center for Advancing Electronics Dresden (cfaed), Technische Universität Dresden, Dresden 01062, Germany

[2]Physical Chemistry, Technische Universität Dresden, Zellescher Weg 19, Dresden 01069, Germany

[3]Dresden Center for Nanoanalysis (DCN), Center for Advancing Electronics Dresden (cfaed), Technische Universität Dresden, Helmholtzstraße 18, Dresden 01069, Germany

[4]Zhejiang Key Laboratory of Advanced Fuel Cells and Electrolyzers Technology, Materials Tech Laboratory for Hydrogen & Energy Storage, Ningbo Institute of Materials Technology and Engineering (NIMTE) of the Chinese Academy of Sciences, Ningbo 315201, China

[5]Max Planck Institute of Microstructure Physics, Weinberg 2, Halle 06120, Germany

[6]Center of Hydrogen Science & State Key Laboratory of Metal Matrix Composites, School of Materials Science and Engineering, Shanghai Jiao Tong University, Shanghai, 200240, China

[7]Leibniz Institute for Solid State and Materials Research (IFW) Dresden e.V., Helmholtzstraße 20, Dresden, 01069, Germany

[8]Department of Inorganic Chemistry, Faculty of Chemical Technology, University of Chemistry and Technology Prague, Technická 5, Prague 6, 16628, Czech Republic

Emails: xinliang.feng@tu-dresden.de; minghao.yu@tu-dresden.de

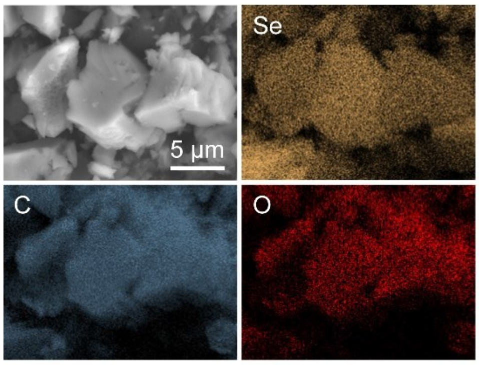

Supplementary Fig. 1 SEM and corresponding EDX elemental mapping images of Se confined in AC.

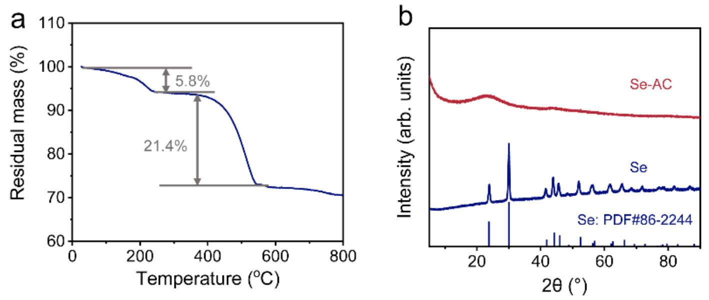

Supplementary Fig. 2 **a** Thermogravimetric analysis of the Se-activated carbon (AC) composite. **b** XRD patterns of pristine Se and the Se-AC composite.

TGA measurement was carried out under an argon atmosphere. The TGA profile of the Se-AC composite shows two mass loss steps, before 200 °C (loss of residual water confined in porous AC, 5.8%) and 550 °C (Se loss, 21.4%), respectively. The Se/AC ratio (*R*) was estimated to be 0.3 according to **equation (S1)**. The Se-AC composite shows no characteristic XRD peaks associated with Se, suggesting the amorphous nature of Se the sample.

$$R = \frac{21.4\%}{1-21.4\%-5.8\%} \approx 0.3 \qquad \textbf{(S1)}$$

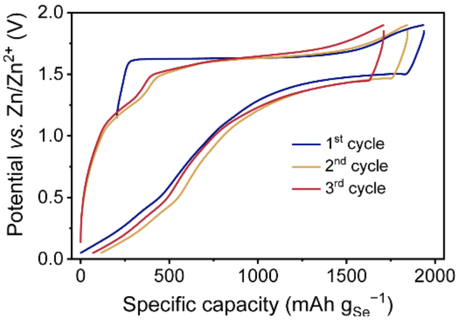

Supplementary Fig. 3 Initial three-cycle GCD profiles of the Se electrode at 0.5 A g_{Se}^{-1}.

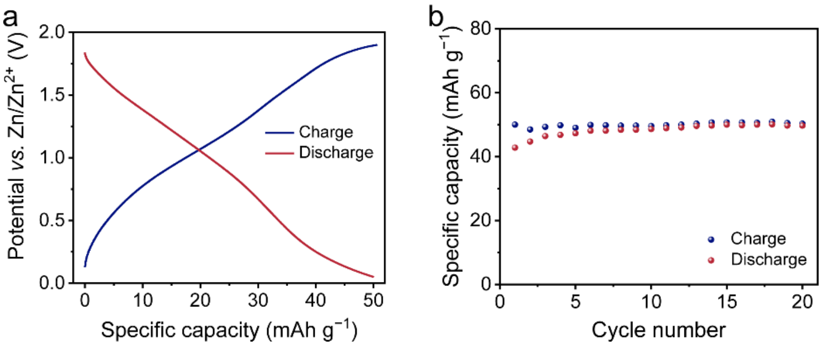

Supplementary Fig. 4 a GCD curve and **b** cycling performance of AC at 50 mA g^{-1} in ZCE. The specific capacity and energy density of active carbon were calculated as 50 mAh g^{-1} and 42.4 Wh kg^{-1}, respectively. To calculate the specific capacity and energy density of Se conversion reaction, we subtracted the contribution from AC to the Se electrode. The electron transfer per Se atom (N) is calculated by **equation (S2)**, where C_e refers to the experimental specific capacity and C_t refers to the theoretical capacity of Se with single electron transfer.

$$N = C_e/C_t \qquad \textbf{(S2)}$$

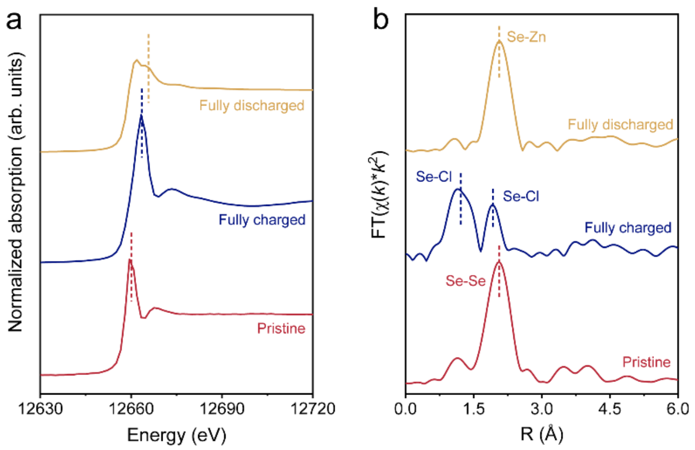

Supplementary Fig. 5 a Se K-edge XANES spectra and **b** FT-EXAFS data in *R*-space of the Se electrode at different charge/discharge states. The XANES spectra were normalized with pre-edge and post-edge.

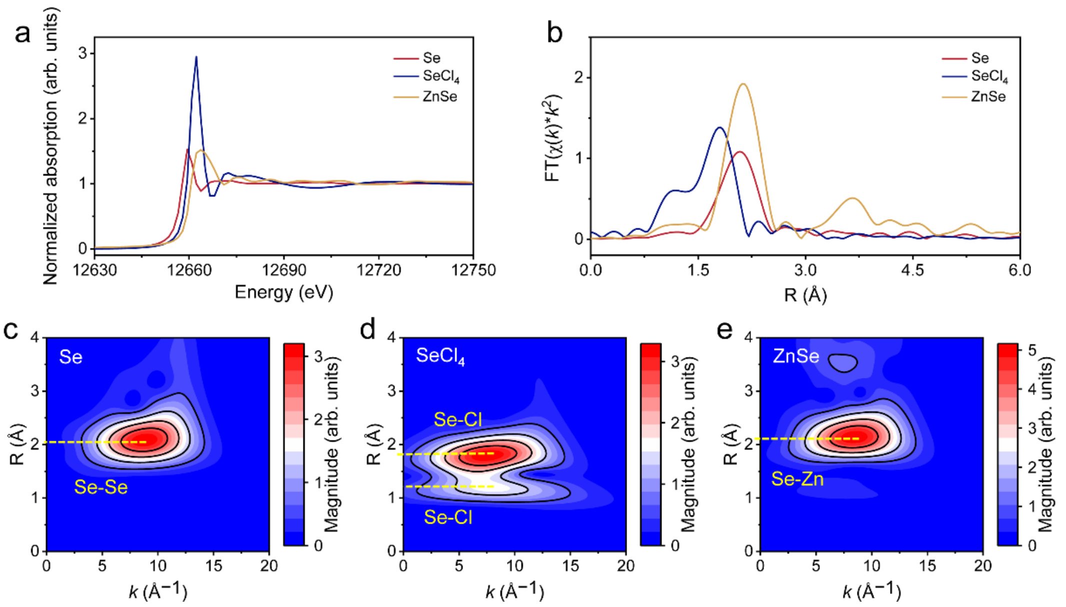

Supplementary Fig. 6 a Se K-edge XANES spectra and **b** FT-EXAFS data in *R*-space of Se, $SeCl_4$, and ZnSe references. Wavelet-transformed Se K-edge EXAFS of **c** Se, **d** $SeCl_4$, and **e** ZnSe references. XANES spectra were normalized with pre-edge and post-edge.

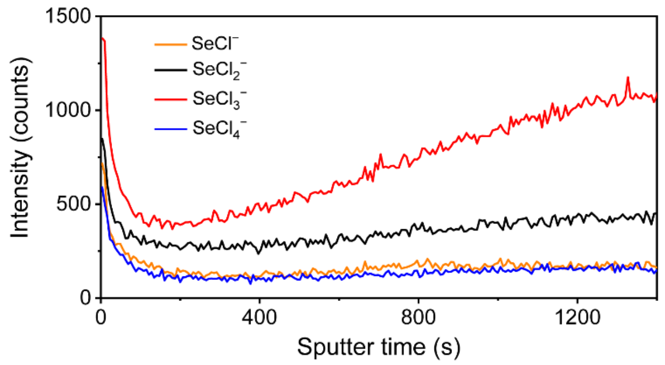

Supplementary Fig. 7 The TOF-SIMS depth profile of the $SeCl_4^-$, $SeCl_3^-$, $SeCl_2^-$, and $SeCl^-$ signals.

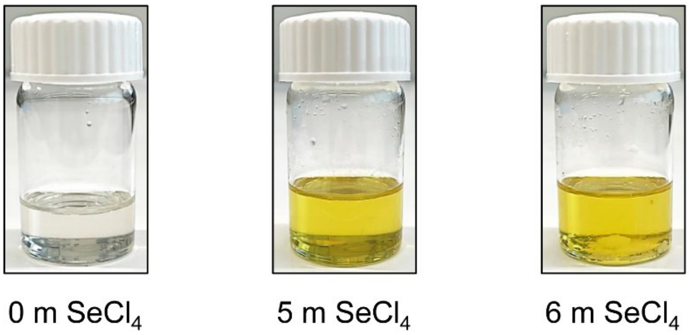

Supplementary Fig. 8 Digital photos of $SeCl_4$ dissolved in 30 m $ZnCl_2$ with varying concentrations.

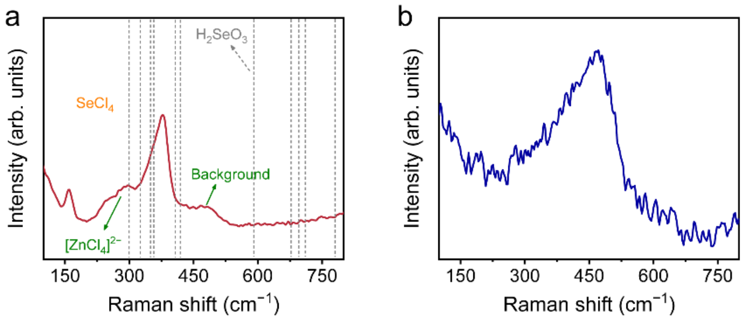

Supplementary Fig. 9 Raman spectra of **a** 5 m $SeCl_4$ dissolved in 30 m $ZnCl_2$ and **b** the glass vessel used to contain the solution.

As shown in **Supplementary Fig. 9a**, the sample presents the characteristic $SeCl_4$ peaks at around 163 cm^{-1} and 375 cm^{-1},[1] along with the peak associated with the $[ZnCl_4]^{2-}$ species[2] and the background peak of the glass vessel (**Supplementary Fig. 9b**). No peaks associated with H_2SeO_3 are detectable[3]. This result indicates that the hydrolysis of $SeCl_4$ to form H_2SeO_3 is suppressed in the highly concentrated $ZnCl_2$ electrolyte due to the reduced reactivity of water.

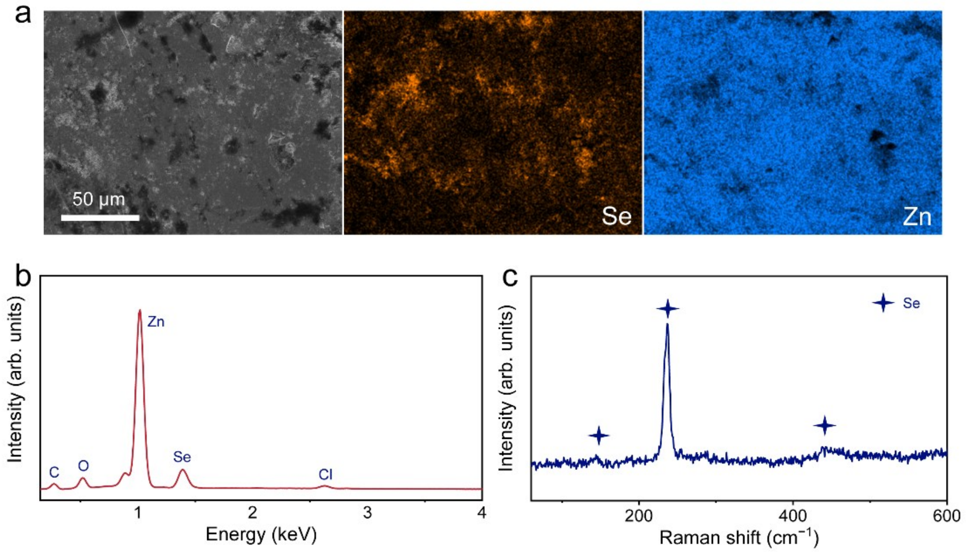

Supplementary Fig. 10 a SEM and corresponding EDX elemental mapping images, **b** EDX, and **c** Raman spectra of the Zn anode in Zn||Se cell using 30 m $ZnCl_2$ + 0.1 m $SeCl_4$ as the electrolyte.

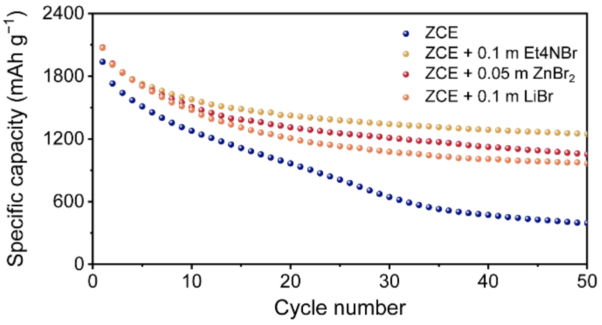

Supplementary Fig. 11 Cycling performance of the Se electrode at 0.5 A g_{Se}^{-1} in ZCE added with different bromide salts.

Different bromide salts exhibited slightly varied effects in stabilizing the Se conversion reaction. However, the cation effect cannot be considered decisive, as all bromide salts demonstrated significant improvements in cycling performance. Among the three bromide salts tested, tetraethylammonium bromide (Et_4NBr) enabled the Zn||Se cell to achieve the best cycling performance. This can be attributed to the bulky nature of Et_4N^+ cations, which may associate with the conversion charging products to form large clusters[4, 5], thus mitigating its dissolution into the electrolyte.

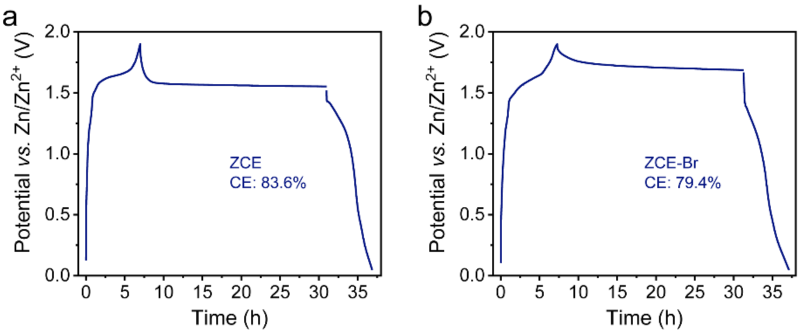

Supplementary Fig. 12 Self-discharge test of the Zn||H-Se cells with **a** ZCE and **b** ZCE-Br. The cells were initially charged to 1.9 V at 0.2 A g_{Se}^{-1}, then kept under open-circuit standing for 24 h, and finally discharged to 0.05 V at 0.2 A g_{Se}^{-1}. The H-Se electrodes were used for the assembly of the Zn||Se cells.

As revealed, both cells retained their original discharge curve shapes after the open-circuit period. The Zn||H-Se cell with ZCE-Br exhibited a slightly lower coulombic efficiency (79.4%) than the cell with ZCE (83.6%), likely due to the reaction of shuttled Br_n^- species with the Zn metal anode. Nevertheless, the effect of Br_n^- on the self-discharge issue is not significant, because only a limited amount of Br_n^- species are generated and can migrate to the anode during the discharge process of the Zn||Se cell.

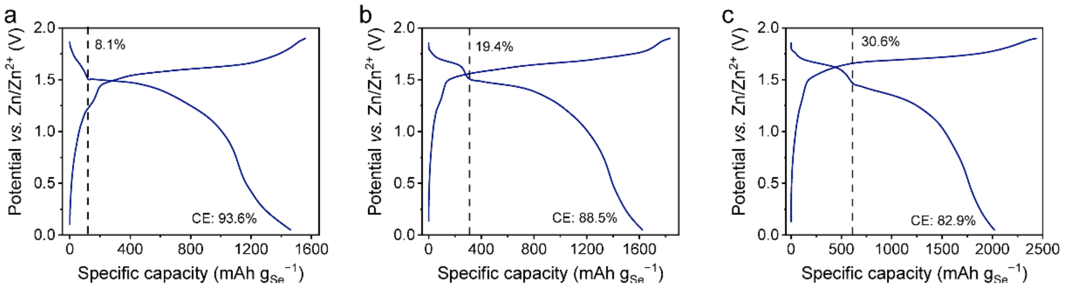

Supplementary Fig. 13 The 2nd GCD profiles of the Zn||H-Se cells with ZCE-Br containing **a** 0.1 m, **b** 0.5 m, and **c** 1 m Et_4NBr at 0.2 A g_{Se}^{-1}. The H-Se electrodes were used for the assembly of the Zn||Se cells.

Increasing the Et_4NBr concentration results in a more pronounced discharge plateau at around 1.7 V, which corresponds to the Br_n^-/Br^- reduction. This observation reflects that higher Et_4NBr concentrations trigger more Br^-/Br_n^- redox reaction in the Zn||Se cell. However, higher Et_4NBr concentrations also lead to lower coulombic efficiency for the Zn||Se cell. That's because excess Br_n^- species generated during charging tend to migrate to the anode side and react with the Zn metal. Thereby, the Et_4NBr concentration must be optimized to minimize the generation of excess Br_n^-. With 0.1 m Et_4NBr, this negative effect could be restricted to minimal, enabling the Zn||Se cell to achieve a coulombic efficiency of 93.6% at the second cycle.

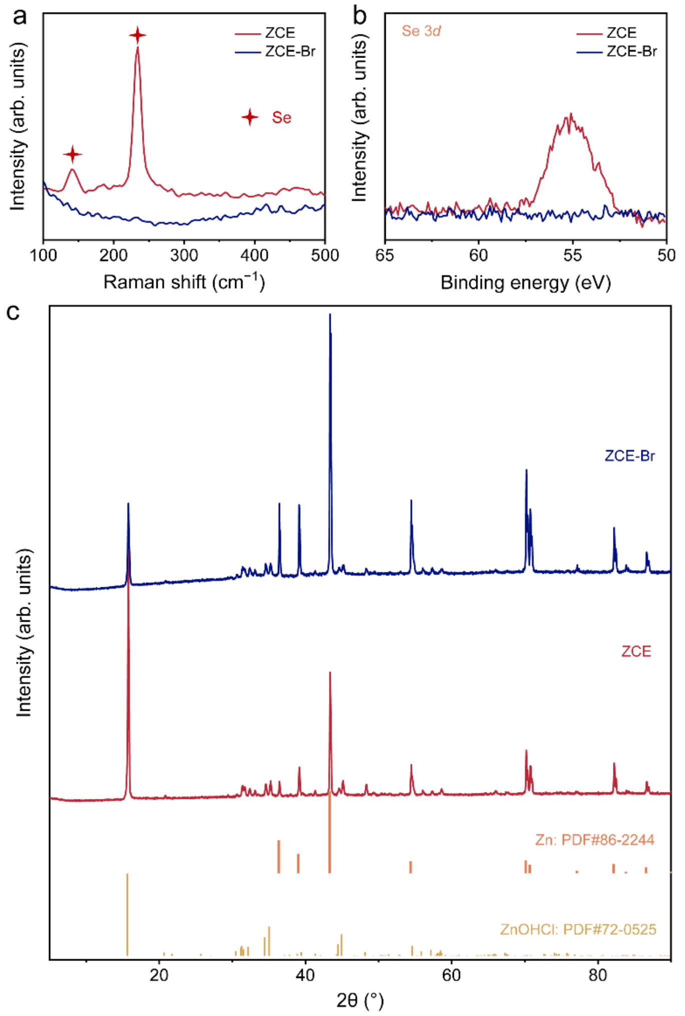

Supplementary Fig. 14 a Raman spectra, **b** XPS spectra, and **c** XRD spectra of the Zn anodes in Zn||Se cells with ZCE and ZCE-Br after 10 cycles at 0.5 A g_{Se}^{-1}.

The Zn anode cycled in ZCE exhibits obvious Se signals, indicating the presence of dead Se. In contrast, the Se signal is barely detectable on the Zn anode cycled in ZCE-Br, verifying the suppression of dead Se formation. The XRD patterns show no characteristic peaks associated with Se on both cycled Zn anodes, implying the low crystallinity or amorphous nature of dead Se on the Zn surface.

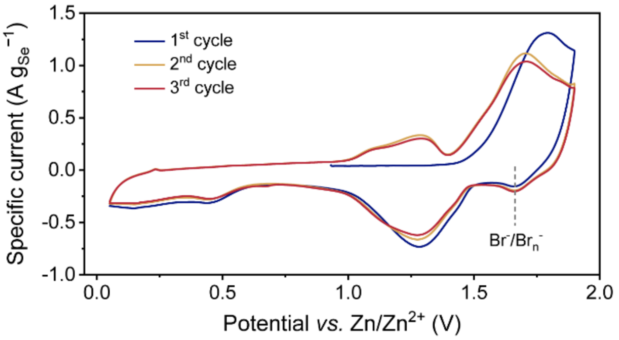

Supplementary Fig. 15 CV profiles of the Zn||Se cells with ZCE-Br at 0.1 mV s^{-1}.

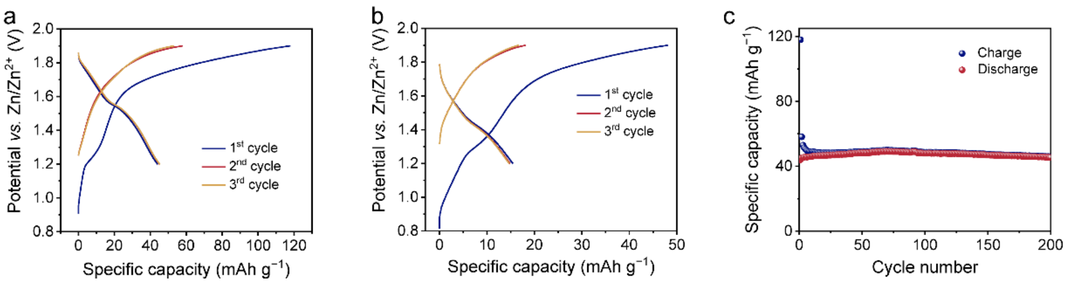

Supplementary Fig. 16 The initial three-cycle GCD profiles of the Zn||AC cell with **a** ZCE-Br and **b** ZCE at 0.2 A g^{-1}. **c** Cycling performance of the Zn||AC cell with ZCE-Br at 0.2 A g^{-1}.

To evaluate the reversibility of Br^{-}/Br_n^{-} redox couple, we assembled Zn||AC cells with ZCE and ZCE-Br. The initial charge cycle of the Zn||AC cell with ZCE-Br exhibits a large irreversible capacity, which can be attributed to the oxidation of poly(ethylene oxide), generating ester species[6] at the cathode/electrolyte solid-solid interface (**Supplementary Fig. 16a**). A similar irreversible capacity was also observed in the Zn||AC cell with ZCE (**Supplementary Fig. 16b**). The slight platform-like shape observed could be attributed to redox-like behavior associated with ion solvation/desolvation process[7-9] or surface functional groups on activated carbon[10-12].cFrom the second cycle onward, the Zn||AC cell with ZCE-Br demonstrates almost identical GCD profiles, indicating the reversible Br^{-}/Br_n^{-} conversion. Moreover, this good reversibility is evidenced by the superior cycling performance of the Zn||AC cell with ZCE-Br (**Supplementary Fig. 16c**).

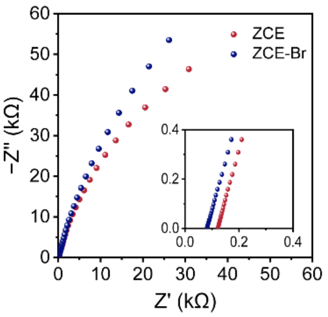

Supplementary Fig. 17 Nyquist plots of ZCE and ZCE-Br.

The ionic conductivities of ZCE and ZCE-Br were measured using Swagelok cells with two stainless-steel rods as current collectors. Electrochemical impedance spectroscopy (EIS) was measured at 25 °C with an amplitude voltage of 5 mV over a frequency range of 100000 ~ 0.1 Hz. The ionic conductivities (σ) of ZCE and ZCE-Br were calculated to be 2.1×10^{-4} S cm^{-1} and 3.1×10^{-4} S cm^{-1}, respectively, based on **equation (S3)**, where d (cm) represents the thickness of the electrolyte, S (cm^2) is the electrolyte area, and R (Ω) is the resistance derived from the Nyquist plots.

$$\sigma = \frac{d}{SR} \qquad \textbf{(S3)}$$

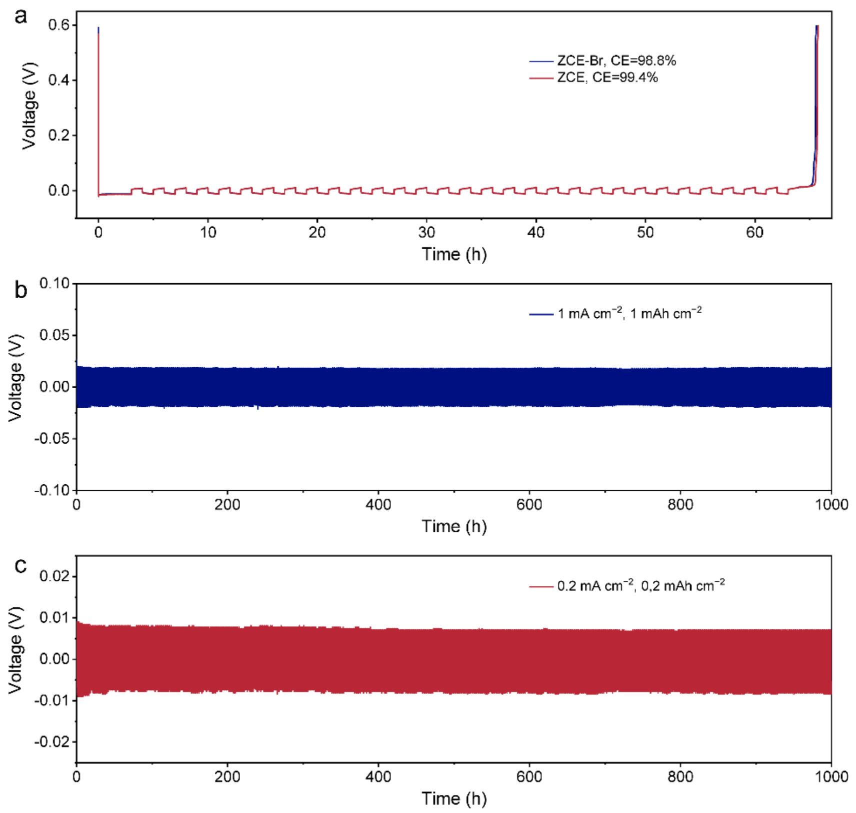

Supplementary Fig. 18 a Coulombic efficiencies (CEs) of Zn||Cu asymmetric cells with ZCE-Br and ZCE measured by the 'Aurbach' method. Voltage profiles of Zn||Zn symmetric cells with ZCE-Br at **b** 1 mA cm^{-2} under a fixed capacity of 1 mAh cm^{-2} and **c** 0.2 mA cm^{-2} under a fixed capacity of 0.2 mAh cm^{-2}.

The CEs were evaluated with the Zn||Cu asymmetric cell using the 'Aurbach' protocol[13], wherein Zn was first deposited on Cu at 0.5 mA cm^{-2} for 3 h, then the cell was cycled at 0.5 mA cm^{-2} and 0.5 mAh cm^{-2} for 30 cycles, and finally Zn was completely stripped from Cu. The CE was calculated by the total stripping capacity divided by the total plating capacity. As displayed in **Supplementary Fig. 18a**, the Zn||Cu cell with ZCE-Br demonstrated a high CE of 98.8 %, which is only slightly lower than the Zn||Cu cell with ZCE. Furthermore, the cycling stability of the Zn||Zn symmetric cell with ZCE-Br was assessed at two different current densities. The cell exhibited stable operation for 1000 hours at 1 mA cm^{-2} and 1 mAh cm^{-2} with a hysteresis voltage of less than 38 mV (**Supplementary Fig. 18b**). Similarly, at 0.2 mA cm^{-2} and 0.2 mAh cm^{-2}, the cell maintained stable with a hysteresis voltage of less than 15 mV (**Supplementary Fig. 18c**).

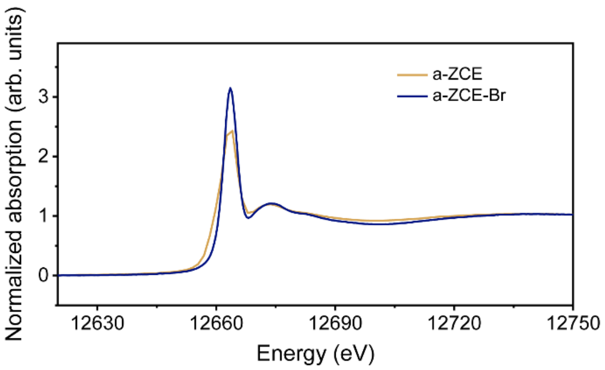

Supplementary Fig. 19 Se K-edge XANES spectra of the fully charged Se electrodes in a-ZCE and a-ZCE-Br. The XANES spectra were normalized with pre-edge and post-edge.

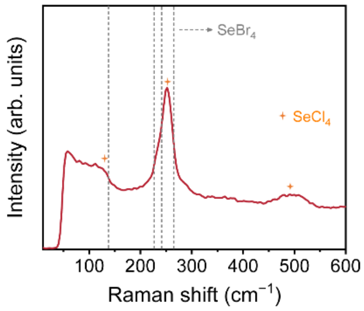

Supplementary Fig. 20 Raman spectrum of the fully charged Se electrode in a-ZCE-Br.

The spectrum exhibits characteristic peaks of $SeCl_4$ at 126 cm^{-1}, 253 cm^{-1}, and a broaden peak ranging from 474 cm^{-1} to 515 cm^{-1}.[1] Meanwhile, peaks associated with $SeBr_4$ are barely detectable[14], indicating $SeCl_4$ is the predominant charge product, while the generation of $SeBr_4$ or $Se(Br_n)_4$ could constitute a minor fraction of the charge product. This statement is also based on the fact that the Cl^- concentration is approximately 300 times higher than the Br^- concentration in the employed ZCE-Br electrolyte.

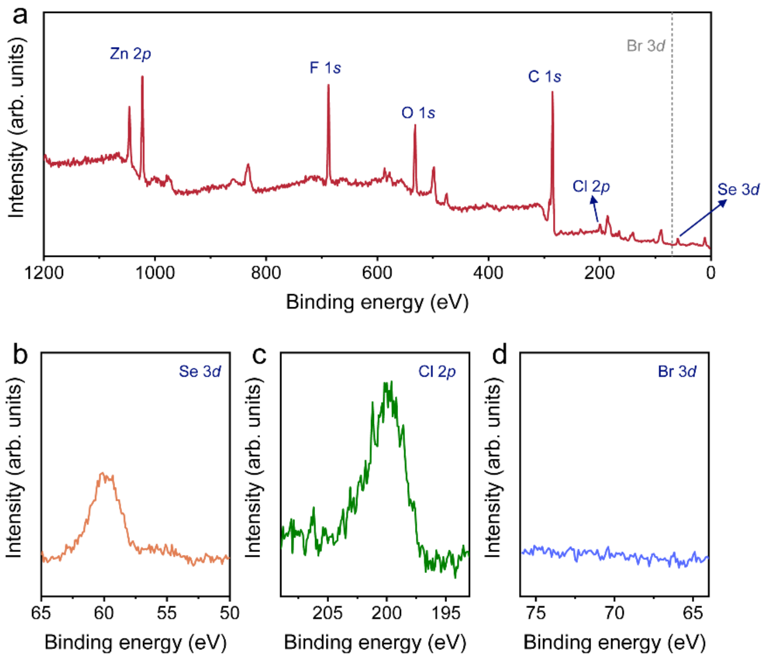

Supplementary Fig. 21 a The survey, **b** Se 3*d*, **c** Cl 2*p*, and **d** Br 3*d* XPS spectra of the fully charged Se electrode in a-ZCE-Br.

The XPS survey spectrum of the fully charged Se electrode after washing is provided in **Supplementary Fig. 21a**. Pronounced Se and Cl signals are observed, whereas the Br signal is not visible. The same conclusion can be derived from the high-resolution Se 3*d* (**Supplementary Fig. 21b**), Cl 2*p* (**Supplementary Fig. 21c**), and Br 3*d* (**Supplementary Fig. 21d**) XPS spectra. These findings further support $SeCl_4$ as the predominant charge product rather than $SeBr_4$.

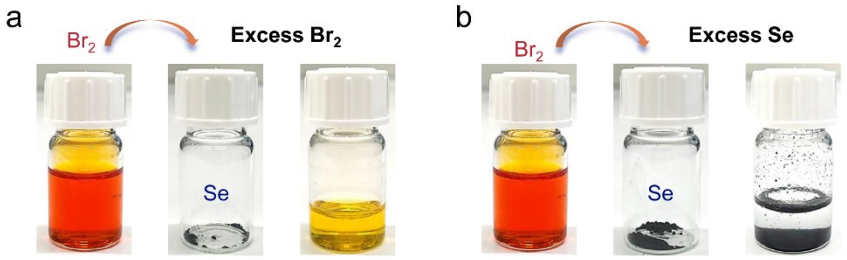

Supplementary Fig. 22 Digital photos showing the reaction of **a** excess Br_2 with Se, as well as **b** Br_2 with excess Se.

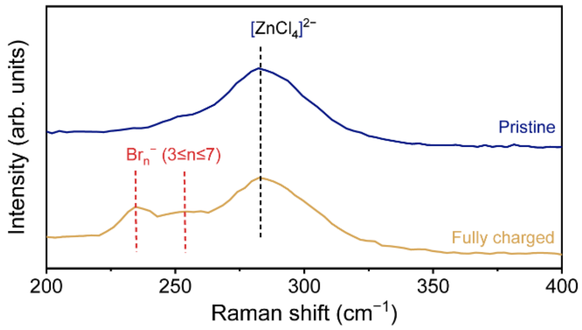

Supplementary Fig. 23 Raman spectra of pristine a-ZCE-Br and a-ZCE-Br in the Zn||Se cell after the first charge cycle.

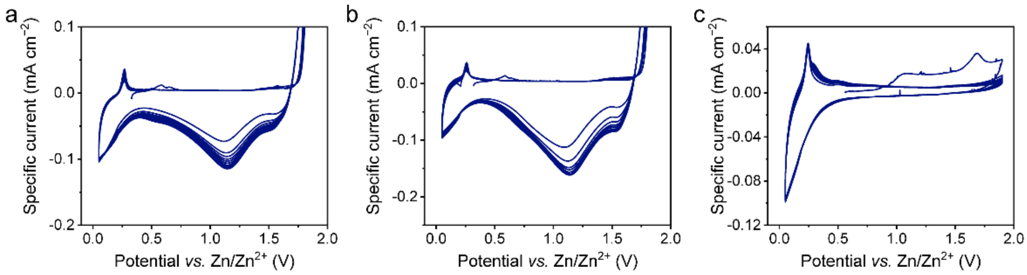

Supplementary Fig. 24 CV curves at 1mV s^{-1} of **a** the Se@Zn||Ti cell with a-ZCE-Br, **b** the Zn||Ti cell with a-ZCE-Br, and **c** the Se@Zn||Ti cell with a-ZCE.

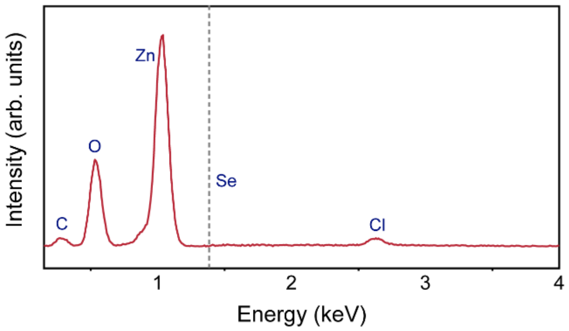

Supplementary Fig. 25 EDX spectrum of the Zn anode of Zn||Se cell after 200 cycles in ZCE-Br.

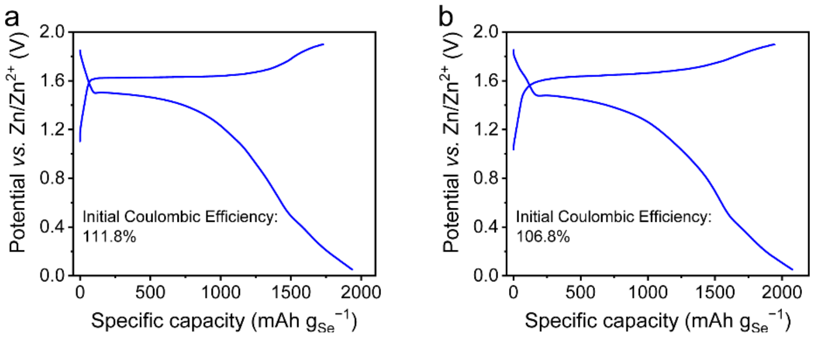

Supplementary Fig. 26 Initial GCD profiles of the Zn||Se cells with **a** ZCE and **b** ZCE-Br at 0.5 A g_{Se}^{-1}.

Supplementary Fig. 26a displays the initial GCD profile of the Zn||Se cell with ZCE. In the initial charge cycle, the specific capacity (1732.9 mAh g_{Se}^{-1}) significantly exceeds the theoretical Se/Se^{4+} capacity (1358.7 mAh g_{Se}^{-1}). This additional capacity is attributed to the irreversible oxidation of poly(ethylene oxide) at the cathode/electrolyte solid-solid interface. In the subsequent discharge cycle, the specific capacity (1937.3 mAh g_{Se}^{-1}) closely aligns with the theoretical Se^{2-}/Se^{0}/Se^{4+} conversion capacity (2038.0 mAh g_{Se}^{-1}), resulting in an initial coulombic efficiency of 111.8%. In the Zn||Se cell with ZCE-Br (**Supplementary Fig. 26b**), both the initial charge (1946.2 mAh g_{Se}^{-1}) and discharge (2077.6 mAh g_{Se}^{-1}) capacities are slightly increased due to the incorporation of the Br^{-}/Br_n^{-} redox couple. The initial coulombic efficiency (106.8%) is comparable to that of the Zn||Se cell with ZCE.

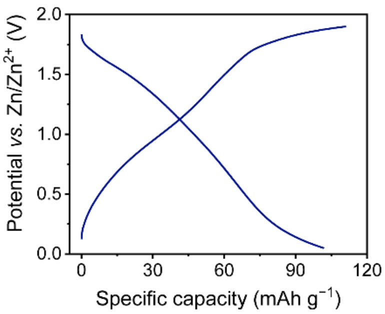

Supplementary Fig. 27 GCD curves of the Zn||Se cell with ZCE-Br.

We assembled a Zn||AC cell with ZCE-Br and derived the specific capacity of the AC electrode (101.8 mAh g^{-1}) from the GCD measurement. This specific capacity includes the contribution from the Br^{-}/Br_n^{-} couple and the capacitive charge storage of AC.

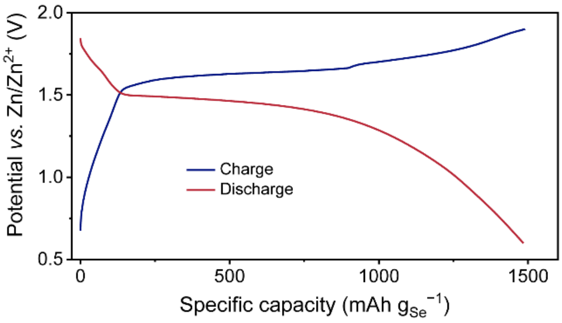

Supplementary Fig. 28 GCD curves of the Zn||Se cell with ZCE-Br in a potential window of 0.6~1.9 V *vs.* Zn/Zn^{2+} at 0.5 A g_{Se}^{-1}.

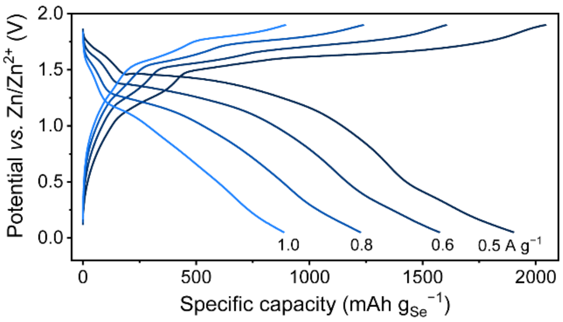

Supplementary Fig. 29 GCD curves of the Zn||Se cell with ZCE-Br at varying specific currents.

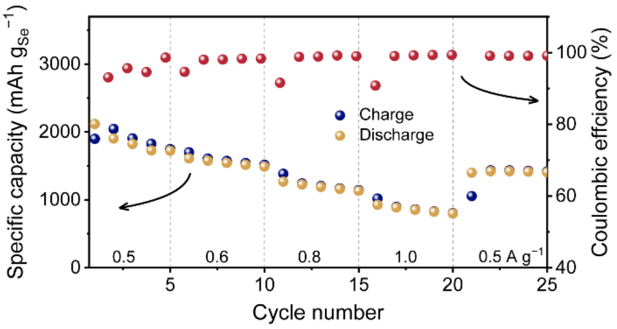

Supplementary Fig. 30 Rate performance of the Zn||Se cell with ZCE-Br.

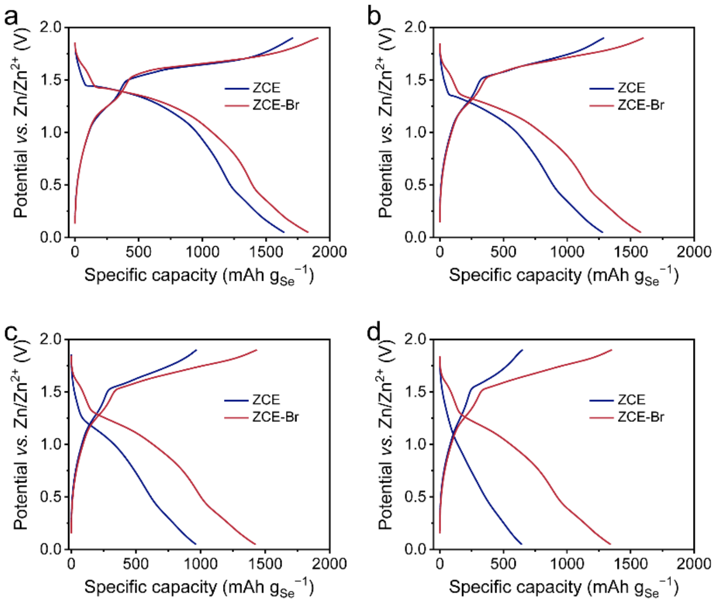

Supplementary Fig. 31 a The 3rd, **b** 10th, **c** 20th, and **d** 30th GCD profiles of Zn||Se cells with ZCE and ZCE-Br at 0.5 A g_{Se}^{-1}.

The increasing polarization observed during cycling can be assigned to structural degradation of the electrode caused by the large volume change that occur during charge and discharge. These volume changes lead to irreversible structural and morphological evolution, including particle pulverization and material breakdown. Such changes result in poor electrical contact, increased charge-transfer resistance, and, consequently, greater polarization. To address this issue, the development of advanced porous hosts with strong confinement capabilities, along with flexible binders possessing robust mechanical properties, is highly desirable and warrants extensive future research efforts.

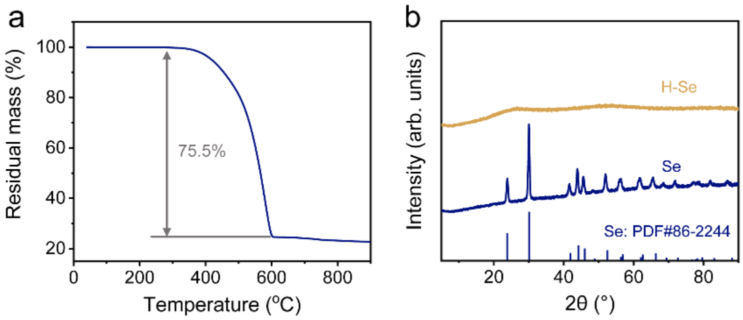

Supplementary Fig. 32 **a** Thermogravimetric analysis H-Se electrode. **b** XRD patterns of pristine Se and H-Se.

The TGA measurement was carried out under an argon atmosphere. The TGA profile of the H-Se shows only the Se loss step (75.5%), as the large Se content occupies the porous space of AC, leaving no room for water retention. The Se/AC ratio (R_H) was estimated to be 3.1 according to **equation (S4)**. H-Se shows no characteristic XRD peaks associated with Se, suggesting the amorphous nature of Se in H-Se.

$$R_H = \frac{75.5\%}{1-75.5\%} \approx 3.1 \qquad \textbf{(S4)}$$

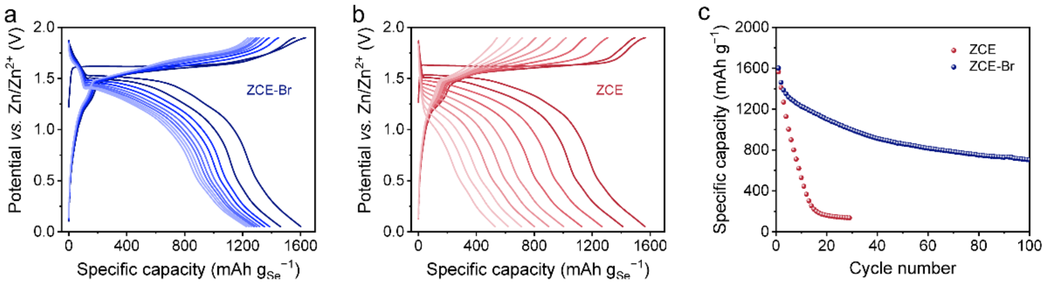

Supplementary Fig. 33 The initial 10-cycle GCD profiles of the H-Se electrode in **a** ZCE-Br and **b** ZCE at 0.2 A g_{Se}^{-1}. **c** Cycling performance of the H-Se electrode in ZCE and ZCE-Br at 0.2 A g_{Se}^{-1}.

In ZCE-Br, the H-Se electrode delivered an initial specific capacity of 1602.8 mAh g_{Se}^{-1} (**Supplementary Fig. 33a**), corresponding to an energy density of 1823 Wh kg_{Se}^{-1}. Moreover, the significant role of Br^-/Br_n^- redox couple in stabilizing the ZnSe/Se/$SeCl_4$ conversion was also demonstrated using the H-Se electrode. In this study, we identified two issues associated with the cycling stability of the Zn||Se cell, namely the dissolution of $SeCl_4$ and subsequent formation of dead Se on the Zn anode. The dead Se formation could accelerate the dissolution of $SeCl_4$ from the cathode, leading to a rapid loss of active cathode materials. The incorporation of the Br^-/Br_n^- redox couple into the Zn||Se cell enables the generated Br_n^- species to act as a dead-Se revitalizer. These species react with the Se passivation layer on the Zn anode, regenerating active Se for cathode reactions. However, our strategy does not entirely prevent the dissolution of $SeCl_4$ from the cathode. Additionally, structural collapse in the cathode may contribute to the continuous capacity loss, particularly due to the substantial volume changes during the conversion reaction (e.g., >500% volume expansion from Se to $SeCl_4$, > 160% volume expansion from Se to ZnSe). To further address the issue, significant future efforts are desired to optimize the system, such as developing advanced porous hosts with strong confinement capabilities and employing coated interphase/functionalized separators to inhibit the dissolution of active Se species.

Supplementary Table 1 Performance comparison with the reported ZAB cathode materials.

Cathode materials	Mass load of active materials (mg cm^{-2})	Active materials/host ratio	Electrolyte	Midpoint voltage (V)	Specific capacity (mAh g^{-1})	Specific energy (Wh kg^{-1})	Ref.
Se (22.7% in Se-AC compound)	1~1.5	0.3	30 m $ZnCl_2$ + 0.1 m Et_4NBr + PEO	~1.03	2077	2138	This work
Se (75.5% in Se-AC compound)	1~1.5	3.1	30 m $ZnCl_2$ + 0.1 m Et_4NBr + PEO	~1.14	1603	1823	This work
α-MnO_2	1~5	/	2 M $ZnSO_4$ + 0.1 M $MnSO_4$	~1.31	285	373	[15]
β-MnO_2	~2	/	3 M $Zn(CF_3SO_3)_2$ + 0.1 M $Mn(CF_3SO_3)_2$	~1.13	225	254	[16]
$K_{0.8}Mn_8O_{16}$	/	/	2 M $ZnSO_4$ + 0.1 M $MnSO_4$	~1.24	320	398	[17]
γ-MnO_2	/	/	1 M $ZnSO_4$	~1.25	285	356	[18]
V_2O_5	~2	4	3 M $Zn(CF_3SO_3)_2$	~0.58	470	273	[19]
V_3O_7•H_2O	/	/	1 M $ZnSO_4$	~0.64	375	240	[20]
$Zn_{0.3}V_2O_5$•1.5H_2O	~2	/	3 M $Zn(CF_3SO_3)_2$	~0.79	426	337	[21]

Calix[4]quinone	2.5~10	/	3 M $Zn(CF_3SO_3)_2$	~1	335	335	22
Para-dinitrobenzene	1.8~2.1	1.5	3 M $Zn(CF_3SO_3)_2$	~0.57	402	230	23
Polyaniline	~1.5	/	1 M $Zn(CF_3SO_3)_2$	~1.1	200	220	24
3, 7-bis(phenylamino)phenothiazin-5-ium iodide	~4	/	2.0 M $ZnSO_4$	~1.1	188	207	25
1,4,5,8,9,12-hexaazatriphenylene-based COFs.	1~1.5	/	2.0 M $ZnSO_4$	~0.84	344	289	26
$Na_3V_2(PO_4)_3$	/	/	0.5 M $Zn(CH_3COO)_2$	~1.1	97	107	27
$FeFe(CN)_6$	~5	/	1 M $Zn(OAc)_2$	~1.3	122	159	28
$CuFe(CN)_6$	~5	/	20 mM $ZnSO_4$	~1.73	53	92	29
$CoFe(CN)_6$	/	/	4 M $Zn(CF_3SO_3)_2$	~1.75	173.4	303	30
I_2	2.36	0.6	30 m $ZnCl_2$	~1.48	612.5	905	31
LF-PLSD	4	/	1 M $Zn(TFSI)_2$	~0.63	1148	725	32
Se	4~10	2.5	4 M $Zn(CF_3SO_3)_2$ + PEO	~1.1	664.7	729	33
Se	~1 and ~5.2	1	1 M $ZnSO_4$	~1.23	611	751	34

TP-Se	~2.2	/	1 M $Zn(OTF)_2$	~1.95	72.9	142	[35]
Te	1~1.5	1.1	30 m $ZnCl_2$ + PEO	~0.84	1223.9	1028	[36]
Te	~1.5	/	1 M $ZnSO_4$	~0.58	419	241	[37]
Te	~1.5	2	30 m $ZnCl_2$	~0.83	802.7	~666	[38]

Supplementary Table 2 The source of the literature data shown in Fig. 5c:

Cathode materials	Mass load of active materials (mg cm^{-2})	Thickness of electrode (μm)	Electrolyte	Electrolyte amount	Temperature (℃)	Cell type	Ref.
α-MnO_2	1~5	/	2 M $ZnSO_4$ + 0.1 M $MnSO_4$	/	/	Coin cell	[15]
β-MnO_2	~2	/	3 M $Zn(CF_3SO_3)_2$ + 0.1 M $Mn(CF_3SO_3)_2$	/	/	Coin cell	[16]
$V_2O_5 \cdot nH_2O$	~1.8	/	3 M $Zn(CF_3SO_3)_2$ + 0.1 M vanadium sol	/	Room temperature	Coin cell	[39]
VO_2	/	/	3 M $Zn(CF_3SO_3)_2$	/	Room	Coin cell	[40]

					temperature		
Calix[4]quinone	2.5~10	~73	3 M $Zn(CF_3SO_3)_2$	Electrolyte impregnated Nafion membrane	/	Coin cell	[22]
Te	1~1.5	/	30 m $ZnCl_2$ + PEO	/	/	Swagelok cell	[36]
Se	4~10	/	4 M $Zn(CF_3SO_3)_2$ + PEO	/	Room temperature	Coin cell	[33]
LF-PLSD	4	~600	1 m $Zn(TFSI)_2$ + 21 m LiTFSI	/	/	Coin cell	[32]
Se	1~1.5	~250	30 m $ZnCl_2$ + 0.1 m Et_4NBr + PEO	Electrolyte impregnated glass fiber membrane	Room temperature	Swagelok cell	This work

Supplementary References

1. Robinson E, Ciruna J. The chlorosulfuric acid solvent system. Part II. The solutes $SeCl_4$ and $TeCl_4$; evidence for the formation of the $SeCl_3^+$ and $TeCl_3^+$ ions. *Can. J. Chem.* **46**, 3197-3200 (1968).
2. Ji X. A perspective of $ZnCl_2$ electrolytes: the physical and electrochemical properties. *eScience* **1**, 99-107 (2021).
3. Torrie B. Raman and Infrared Spectra of Na_2SeO_3, $NaHSeO_3$, H_2SeO_3, and $NaH_3(SeO_3)_2$. *Can. J. Phys.* **51**, 610-615 (1973).
4. Dai C*, et al.* Fast constructing polarity-switchable zinc-bromine microbatteries with high areal energy density. *Sci. Adv.* **8**, eabo6688 (2022).
5. Wang C*, et al.* Visualizing and Understanding the Ionic Liquid-Mediated Polybromide Electrochemistry for Aqueous Zinc-Bromine Redox Batteries. *Nano Lett.* **24**, 13796-13804 (2024).
6. Qiu J*, et al.* Enabling stable cycling of 4.2 V high-voltage all-solid-state batteries with PEO-based solid electrolyte. *Adv. Funct. Mater.* **30**, 1909392 (2020).
7. Ge K, Shao H, Raymundo-Piñero E, Taberna P-L, Simon P. Cation desolvation-induced capacitance enhancement in reduced graphene oxide (rGO). *Nat. Commun.* **15**, 1935 (2024).
8. Hu L, Guo D, Feng G, Li H, Zhai T. Asymmetric behavior of positive and negative electrodes in carbon/carbon supercapacitors and its underlying mechanism. *J. Phys. Chem. C* **120**, 24675-24681 (2016).
9. Urita K, Ide N, Isobe K, Furukawa H, Moriguchi I. Enhanced electric double-layer capacitance by desolvation of lithium ions in confined nanospaces of microporous carbon. *ACS nano* **8**, 3614-3619 (2014).
10. Hu YR, Dong XL, Zhuang HK, Yan D, Hou L, Li WC. Introducing Electrochemically Active Oxygen Species to Boost the Pseudocapacitance of Carbon-based Supercapacitor. *ChemElectroChem* **8**, 3073-3079 (2021).
11. Qiu C, Jiang L, Gao Y, Sheng L. Effects of oxygen-containing functional groups on carbon materials in supercapacitors: A review. *Mater. Design* **230**, 111952 (2023).
12. Liu F, Xue D. An electrochemical route to quantitative oxidation of graphene frameworks with controllable C/O ratios and added pseudocapacitances. *Chem. Eur. J.* **19**, 10716-10722 (2013).
13. Chen C, Zhang J, Hu B, Liang Q, Xiong X. Dynamic gel as artificial interphase layer for ultrahigh-rate and large-capacity lithium metal anode. *Nat. Commun.* **14**, 4018 (2023).
14. Murchie MP, Passmore J, White PS. The characterisation and X-ray crystal structure of pentabromodiselenium hexafluoroarsenate, $Se_2Br_5AsF_6$; some thermodynamic considerations and the nonexistence of $Se_2I_5AsF_6$. *Can. J. Chem.* **65**, 1584-1593 (1987).
15. Pan H*, et al.* Reversible aqueous zinc/manganese oxide energy storage from conversion reactions. *Nat. Energy* **1**, 16039 (2016).
16. Zhang N*, et al.* Rechargeable aqueous zinc-manganese dioxide batteries with high energy and power densities. *Nat. Commun.* **8**, 405 (2017).
17. Fang G*, et al.* Suppressing manganese dissolution in potassium manganate with rich oxygen defects engaged high-energy-density and durable aqueous zinc-ion battery. *Adv.*

Funct. Mater. **29**, 1808375 (2019).
18. Alfaruqi MH*, et al.* Electrochemically induced structural transformation in a γ-MnO_2 cathode of a high capacity zinc-ion battery system. *Chem. Mater.* **27**, 3609-3620 (2015).
19. Zhang N*, et al.* Rechargeable aqueous Zn–V_2O_5 battery with high energy density and long cycle life. *ACS Energy Lett.* **3**, 1366-1372 (2018).
20. Kundu D, Hosseini Vajargah S, Wan L, Adams B, Prendergast D, Nazar LF. Aqueous vs. nonaqueous Zn-ion batteries: consequences of the desolvation penalty at the interface. *Energy Environ. Sci.* **11**, 881-892 (2018).
21. Wang LL, Huang KW, Chen JT, Zheng JR. Ultralong cycle stability of aqueous zinc-ion batteries with zinc vanadium oxide cathodes. *Sci. Adv.* **5**, 10 (2019).
22. Zhao Q*, et al.* High-capacity aqueous zinc batteries using sustainable quinone electrodes. *Sci. Adv.* **4**, 10 (2018).
23. Song Z*, et al.* Anionic co-insertion charge storage in dinitrobenzene cathodes for high-performance aqueous zinc–organic batteries. *Angew. Chem. Int. Ed.* **61**, e202208821 (2022).
24. Wan F, Zhang L, Wang X, Bi S, Niu Z, Chen J. An aqueous rechargeable zinc-organic battery with hybrid mechanism. *Adv. Funct. Mater.* **28**, 1804975 (2018).
25. Wang N*, et al.* Molecular tailoring of an n/p-type phenothiazine organic scaffold for zinc batteries. *Angew. Chem. Int. Ed.* **60**, 20826-20832 (2021).
26. Wang W*, et al.* Molecular engineering of covalent organic framework cathodes for enhanced zinc-ion batteries. *Adv. Mater.* **33**, 2103617 (2021).
27. Li G*, et al.* Towards polyvalent ion batteries: A zinc-ion battery based on NASICON structured $Na_3V_2(PO_4)_3$. *Nano Energy* **25**, 211-217 (2016).
28. Liu Z, Pulletikurthi G, Endres F. A Prussian blue/zinc secondary battery with a bio-Ionic liquid-water mixture as electrolyte. *ACS Appl. Mater. Interfaces* **8**, 12158-12164 (2016).
29. Trocoli R, La Mantia F. An aqueous zinc-ion battery based on copper hexacyanoferrate. *ChemSusChem* **8**, 481-485 (2015).
30. Ma L*, et al.* Achieving high-voltage and high-capacity aqueous rechargeable zinc Ion battery by incorporating two-species redox reaction. *Adv. Energy Mater.* **9**, 1902446 (2019).
31. Liang G*, et al.* Development of rechargeable high-energy hybrid zinc-iodine aqueous batteries exploiting reversible chlorine-based redox reaction. *Nat. Commun.* **14**, 1856 (2023).
32. Zhao Y*, et al.* Initiating a reversible aqueous Zn/sulfur battery through a "Liquid Film". *Adv. Mater.* **32**, e2003070 (2020).
33. Ma L*, et al.* Electrocatalytic Selenium Redox Reaction for High-Mass-Loading Zinc-Selenium Batteries with Improved Kinetics and Selenium Utilization. *Adv. Energy Mater.* **12**, 2201322 (2022).
34. Chen Z*, et al.* Zinc/selenium conversion battery: a system highly compatible with both organic and aqueous electrolytes. *Energy Environ. Sci.* **14**, 2441-2450 (2021).
35. Chen Z*, et al.* Anion chemistry enabled positive valence conversion to achieve a record high-voltage organic cathode for zinc batteries. *Chem* **8**, 2204-2216 (2022).
36. Du J*, et al.* A High-Energy Tellurium Redox-Amphoteric Conversion Cathode

Chemistry for Aqueous Zinc Batteries. *Adv. Mater.*, 2313621 (2024).
37. Chen Z*, et al.* Aqueous zinc-tellurium batteries with ultraflat discharge plateau and high volumetric capacity. *Adv. Mater.* **32**, e2001469 (2020).
38. Chen Z*, et al.* Tellurium with Reversible Six-Electron Transfer Chemistry for High-Performance Zinc Batteries. *J. Am. Chem. Soc.* **145**, 20521-20529 (2023).
39. Yan M*, et al.* Water-lubricated intercalation in $V_2O_5 \cdot nH_2O$ for high-capacity and high-rate aqueous rechargeable zinc batteries. *Adv. Mater.* **30**, 1703725 (2018).
40. Ding J*, et al.* Ultrafast Zn^{2+} intercalation and deintercalation in vanadium dioxide. *Adv. Mater.* **30**, e1800762 (2018).
